# Lessons From a Complex Case of Calcific Constrictive Pericarditis: A Case Report

**DOI:** 10.1155/cric/5514172

**Published:** 2025-05-09

**Authors:** Varun Kasula, Vikram Padala, Jagroop Doad, Hassan Awais, Vinod Chaubey, Aditya Sood, Lauren Golden

**Affiliations:** ^1^Department of Cardiology, Campbell University School of Osteopathic Medicine, Lillington, North Carolina, USA; ^2^Department of Cardiology, Lewis Katz School of Medicine at Temple University, Philadelphia, Pennsylvania, USA; ^3^Department of Cardiology, Conway Medical Center, Conway, South Carolina, USA

**Keywords:** calcific constrictive pericarditis, diastolic dysfunction, pericardial calcification, pericardiectomy, septal bounce

## Abstract

Despite advances in imaging and diagnostics, calcific constrictive pericarditis (CCP) remains a rare and challenging entity, often masquerading as other cardiopulmonary conditions, leading to delayed diagnosis. We present a 70-year-old male with a history of heart failure, atrial fibrillation (AF), cirrhosis, chronic obstructive pulmonary disease (COPD), and prior pleural effusion, who was admitted with acute hypoxic respiratory failure and AF with rapid ventricular response (RVR). Imaging revealed extensive pericardial calcifications, leading to a diagnosis of CCP. The patient's clinical course was marked by refractory hypotension, altered mental status, and progressive cardiohepatic syndrome. Given his high surgical risk, he was managed conservatively and transitioned to palliative care. This case underscores the diagnostic and therapeutic challenges of CCP, particularly in patients with complex comorbidities where surgical intervention is not feasible. It highlights the need for early recognition and individualized management strategies to optimize outcomes in this challenging subset of patients.

## 1. Introduction

Pericarditis is inflammation of the pericardium, which is a fibrous, avascular sac surrounding the heart that supports normal cardiac functioning, and is particularly important in optimal diastolic function [1]. Constrictive pericarditis (CP) occurs when this inflammation causes thickening, fibrosis, stiffening, and potentially calcification of the pericardium. Constriction of the myocardium disrupts diastolic filling and decreases cardiac output. Pericardial calcification may occur as a result of ongoing inflammation and can either be asymptomatic or contribute to constriction of the myocardium. CP can lead to heart failure, pulmonary edema, pleural effusion [2], ascites precox [3], cirrhosis, and secondary intestinal lymphangiectasia [4]. Clinical manifestations include dyspnea, Kussmaul sign (JVD during inspiration), orthostasis, and pleuritic chest pain.

Common etiologies of CP are viral infection, chest radiation, and cardiac surgery; however, most cases are described as idiopathic. Tuberculosis accounted for nearly half of US CP cases in the past but is now only common in developing nations [5]. Less common etiologies include uremia, malignancy, asbestos exposure, and trauma. Pericardial calcification most commonly occurs following trauma, purulent pericarditis, and acute pericarditis or pericardial effusions associated with malignancy and connective tissue disease [6].

Evaluation of pericardial calcification involves using computed tomography (CT) or magnetic resonance imaging (MRI) to visualize the affected tissue. Echocardiography and heart catheterization are used to assess the impact of constrictive pathology on cardiac function. Surgical pericardiectomy is the gold standard treatment with a high success rate; however, conservative therapy should be trialed initially especially when transient CP is suspected. Medical management involves anti-inflammatory agents like steroids, and when care is palliative, diuretics can be used [5].

In this case, we report on a 70-year-old male with a complex medical history who was diagnosed with calcific CP during his lengthy hospital stay. The case highlights the possible etiologies as well as the diagnostic and management challenges posed by this rare but serious condition.

## 2. Case Presentation

**Figure 1:**
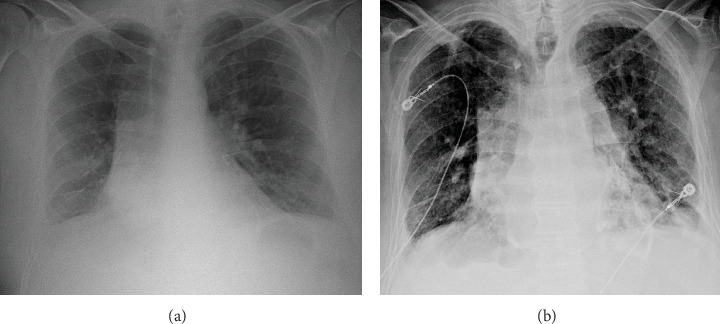
Comparison of CXRs from 2017 (a) and 2024 (b) demonstrating the relatively recent development of pericardial calcifications.

A 70-year-old male with a complex past medical history including paroxysmal AF, combined heart failure with reduced and preserved ejection fraction, cirrhosis, chronic obstructive pulmonary disease (COPD), former heavy tobacco use, former alcohol use disorder, hypothyroidism, and a history of loculated pleural effusion, presented to the hospital with a 2-week history of sharp bilateral back pain and generalized muscle pains as well as a 4-day history of shortness of breath and palpitations. In the ED, the patient was found to be in acute hypoxic respiratory failure with an SpO_2_ of 83% that was responsive to a short course of BiPAP followed by 2 L of O_2_ via nasal cannula. He also was notable for AF with a RVR of 142 that was responsive to IV Lopressor. The blood pressure was stable at 109/72 mmHg. The physical examination revealed diffuse coarse lung sounds, tachypnea, irregular heart rhythm, and significant bilateral lower extremity pitting edema. Initial CXR revealed extensive curvilinear opacities along the pericardial contour, suggestive of calcification—a notable new finding compared to a prior CXR from 2017, which showed no evidence of calcification (Figure 1).

**Figure 2:**
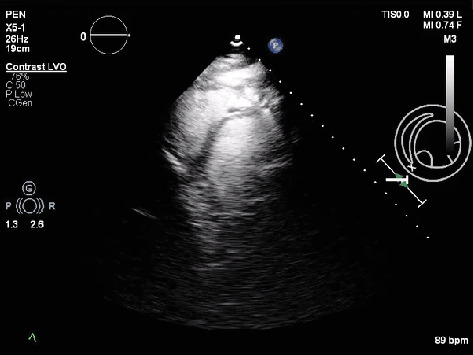
Echocardiogram showing septal bounce (short axis view).

**Figure 3:**
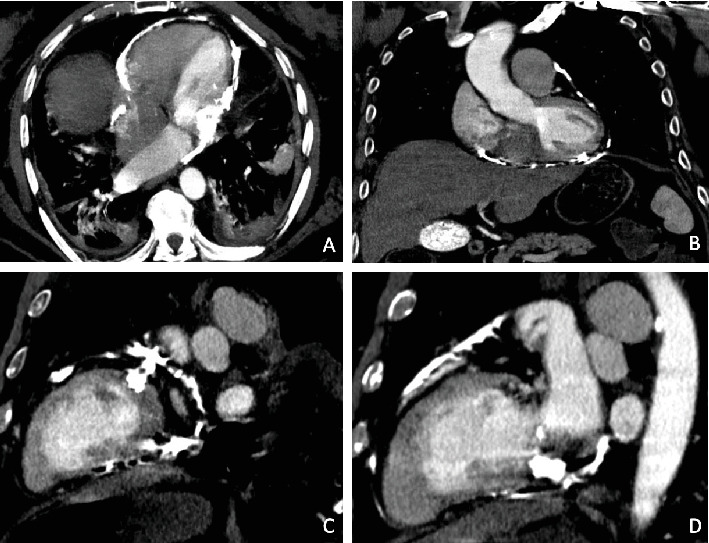
CT thorax with contrast showing pericardial calcifications. (A) Axial view, (B) coronal view, (C) sagittal view showing posterolateral aspects of the heart, and (D) sagittal view showing anteroseptal aspects of the heart.

Patient was admitted directly to the ICU. Initial laboratory findings showed mild hyponatremia, hypochloremia, mild transaminitis, and coagulopathy (PT of 39.7 and INR of 3.42) suggesting hepatic dysfunction. CT abdomen/pelvis suggested the presence of pericardial calcifications, prompting a further workup and a cardiology consultation. The patient underwent tuberculosis screening via a PPD test, which was read as negative with no induration observed at 48–72 h. He also denied any prior history of tuberculosis infection. Echocardiogram revealed an EF of 45%, increased echogenicity of the pericardium, increased septal bounce during diastole (Figure 2), and bowing of the interventricular cavity into the LV. The diagnosis was confirmed by CT thorax, revealing prominent pericardial calcifications consistent with calcific CP (Figure 3).

Throughout his hospital course, the patient experienced fluctuations in his clinical status, including episodes of hypotension, AF with RVR, and altered mental status, which were primarily managed through adjustments to his diuretic, beta-blocker, and lactulose regimen, respectively. Despite these interventions, he continued to exhibit signs of cardiohepatic syndrome such as treatment-resistant shortness of breath, abdominal distention and pain, and pitting lower extremity edema. Then, 1 week after admission, an ABG revealed a pH of 7.34, pCO_2_ of 73.2, and HCO_3_ of 40.3 indicating hypercapnic respiratory acidosis with compensated metabolic alkalosis compensation.

Due to the presence of his multiple comorbidities and the inherent high-risk associated with pericardial stripping—an open surgery that requires a median sternotomy—the decision was made to manage the patient conservatively with a palliative consult. The patient's complex presentation and hospital course highlighted the interplay between his multiple chronic conditions, with calcific CP likely playing a central role in his acute decompensation.

## 3. Discussion

### 3.1. Etiology

Pericarditis involves inflammation of the pericardial sac [7], whereas CP is often a sequela of chronic inflammation and/or scarring of the pericardium [8]. The diagnosis of pericarditis often requires 2 of 4 of the following to be present: a characteristic pericardial chest pain, a pericardial friction rub, widespread ST-elevation and/or PR-depression, and a new or increasing nontrivial pericardial effusion [9, 10]. Generally, the leading cause of pericarditis worldwide is tuberculosis, whereas in the United States, it is often postviral or idiopathic in origin [11]. Other rare causes reported in the literature often include malignancy, connective tissue disorders, trauma, radiation, sarcoidosis, asbestosis, and uremia [12]. In certain cases, such as our patient, a pinpoint etiology can be difficult to elucidate and a further history into its multifactorial causes is warranted.

### 3.2. Pathophysiology of Calcification in CP

Pericardial calcification can result from two primary mechanisms: dystrophic and metastatic calcification [13]. Dystrophic calcification typically occurs in areas of tissue damage, inflammation, necrosis, or hypoxia [13, 14]. With regards to the pericardium, calcification is most often seen in conditions like chronic CP, trauma, chest radiation, or after cardiac interventions. In contrast, metastatic calcification arises from systemic metabolic disturbances that dysregulate calcium metabolism. It is typically seen in conditions such as chronic kidney disease, hyperparathyroidism, or prolonged hypercalcemia [15, 16].

Our patient had multiple risk factors suggesting dystrophic calcification as the most likely mechanism underlying his CCP. First, his long-standing history of COPD and presentation with acute respiratory failure, along with an ABG revealing hypercapnic respiratory acidosis, indicate chronic CO_2_ retention and an underlying hypoxic state, despite not using home oxygen. Additionally, his difficult-to-control atrial fibrillation likely exacerbated diastolic dysfunction by inducing a systemic proinflammatory state, promoting myocardial inflammation and interstitial fibrosis [17]. His elevated CRP level of 8.85 further suggests an acute-on-chronic inflammatory state, potentially accelerating dystrophic calcification. Interestingly, his 45-year career in the automotive parts industry may have exposed him to asbestos, especially during the first half of his career [18]. CT thorax displayed pleural lesions suspicious of nodules or consolidation in our patient which is notable because asbestosis exposure to automobile mechanics has been associated with the development of pleural plaques in a dose-dependent fashion [19]. On rare occasions, asbestosis has been shown to induce pericardial effusion and lead to CP [20, 21]. Lastly, trauma has long been recognized as a potential cause of CP [21, 22], but diagnosis is often difficult due to the variability in clinical presentation. For example, the average time to surgical operation has been reported in certain patients to vary from 3 to up to 20 years [23]. Our patient experienced a left chest wall contusion secondary to a “bad fall” while at work 7 years prior to this admission in 2017—a traumatic incident that may have led to pericardial fibrosis and eventually dystrophic calcification. This hypothesis is supported by the comparison of serial chest radiographs (Figure 1), which demonstrate the absence of pericardial calcifications in 2017 but clear evidence of extensive pericardial calcifications in 2024. The lack of calcifications before the traumatic injury and their relatively recent development render a post-traumatic etiology highly plausible.

We believe metastatic calcification is less likely responsible for this patient's pericardial calcifications, primarily because his corrected calcium levels were consistently normal or borderline low and he had no signs of renal disease [16]. Phosphate levels were not checked; however, our patient did display metabolic disturbances that may have indirectly predisposed him to metastatic calcium deposition. The ABG revealed that the patient had likely been living in a state of chronic respiratory acidosis with a compensatory metabolic alkalosis. Metabolic alkalosis has been associated with calcium dysregulation and enhanced soft tissue calcium deposition. The patient's low chloride levels also suggest a predisposition to an alkalotic state, further promoting metastatic calcification [15, 24–26].

### 3.3. Imaging

Imaging plays a crucial role in the diagnosis and workup of calcific CP. CT thorax with contrast confirmed the presence of prominent and diffuse pericardial calcifications (Figure 3), which are heavily suggestive of calcific CP [27, 28]. The findings of bilateral pleural effusions and hepatic congestion on CT further supported the diagnosis, indicating systemic consequences of CP-induced diastolic dysfunction.

The gold standard imaging tool for diagnosing CP is transthoracic echocardiography (TTE). It often reveals a septal bounce, which occurs during the late filling phase of diastole [29]. Continued atrial contraction into ventricles whose expansion is limited by a stiffened pericardium eventually leads to a rebound bounce effect by the interventricular septum. In our patient, the echocardiogram showed an increased septal bounce (Figure 2) and an echo-bright and thickened pericardium, consistent with calcification. Bowing of the interventricular septum into the left ventricle was also noted, which resembles the noncompliant pericardium causing the right ventricle, under higher pressure, to push the septum into the left ventricle [30].

In CP, both septal bounce and septal bowing are accentuated with inspiration as the decreased intrathoracic pressure leads to increased venous return [30, 31]. This respiratory variation is important in distinguishing CP from restrictive cardiomyopathy (RCM), which is characterized by stiff, noncompliant ventricles and diastolic dysfunction. The myocardium, rather than the pericardium, is affected in RCM. As a result, the ventricles are affected symmetrically and respiration-dependent abnormalities in septal motion are not typically seen [32, 33]. Instead, RCM classically presents with biatrial dilation and nondilated ventricles on TTE. Pulsed-wave Doppler mode can also help differentiate CP from RCM, as RCM is characterized by a significantly elevated *E*/*e*′ ratio, whereas CP typically shows a normal or mildly elevated ratio [31, 33]. This reflects the elevated filling pressure and poor myocardial relaxation in RCM. Although the atrial pressure may also be increased in CP (*E*), myocardial relaxation (*e*′) is relatively preserved.

Heart catheterization is often utilized to confirm the diagnosis of CP, revealing characteristic hemodynamic findings such as equalization of right and left ventricular pressures during diastole [34]. It can also display a pathognomonic “square root sign” on the pressure tracings, which reflects rapid early filling followed by a mid-diastolic plateau. In our patient, the decision to forego catheterization was based on confirmatory noninvasive diagnostic findings from CT and TTE, along with concerns regarding the patient's frailty, advanced age, and comorbidities, making an invasive procedure a less favorable option.

### 3.4. Management

The treatment of choice for CP is often pericardiectomy which usually involves the complete removal of the anterior and diaphragmatic pericardium [35]. A recent study found no significant difference in mortality between pericardiectomy and medical management in patients with relapsing pericarditis. However, patients who received pericardiectomy had a decreased relapse rate compared to those that received medical management [12]. Even with pericardiectomy, postoperative mortality rates can approach 15% with the most significant risk factors being prior cardiac intervention, baseline CKD with dialysis, and prior mediastinal radiation exposure [36]. Notably, the presence of pericardial calcifications has not been shown to have an effect on early survival [37]. Several factors made our patient a poor surgical candidate, including his advanced age, complex medical history, and episodes of hypotension and altered mental status, all of which indicated a limited hemodynamic reserve.

Additionally, his MELD score of 25 at admission signified a high risk of postoperative complications and poor surgical outcomes in CP patients [38]. Given these considerations, medical management was deemed the most appropriate course of action.

## 4. Conclusion

This case highlights the complex interplay between calcific CP and multiple chronic conditions such as heart failure, atrial fibrillation, chronic cirrhosis, and COPD. In patients with advanced age and significant comorbidities, the decision to pursue conservative management over surgical intervention can be difficult but often necessary. CaCP, though rare, can lead to substantial morbidity by impairing diastolic filling and cardiac function, ultimately contributing to systemic complications such as cardiohepatic syndrome. Early recognition through advanced imaging and a multidisciplinary approach is essential for optimizing patient outcomes, particularly for those who are poor surgical candidates. This case emphasizes the importance of considering individualized treatment strategies that consider not only the underlying pathology but also the patient's overall clinical status, comorbidities, and quality of life.

## Data Availability

Data sharing is not applicable to this article as no datasets were generated or analyzed during the current study.
